# The DEPRE’5 study: pragmatic, multicentre, five-arm, parallel-group randomised controlled trial with blinded assessment to compare treatment strategies in major depression after a failed selective serotonin reuptake inhibitor treatment

**DOI:** 10.1192/bjp.2025.13

**Published:** 2025-11

**Authors:** Víctor Pérez, Dolors Puigdemont, Javier de Diego-Adeliño, Matilde Elices, Itziar Leal, Maria Cabello, Roberto Rodriguez-Jimenez, Miguel Ángel Álvarez-Mon, Lorena García-Fernández, Eduardo José Aguilar García-Iturrospe, Maria José Escartí, Angel Luis Montejo, José Manuel Montes, Judith Usall, Ascensión Gallego-Nogueras, Elena Lujan, Raquel López-Carrilero, Ana González-Pinto, Agurtzane Ortiz-Jauregui, Jordi Blanch, Mikel Urretavizcaya, Francesc Colom, Javier García-Campayo, José Luis Ayuso-Mateos

**Affiliations:** Instituto de Salud Mental, Hospital del Mar, Barcelona, Spain; Hospital del Mar Medical Research Institute (IMIM), Barcelona, Spain; Department of Medicine and Life Sciences, Universitat Pompeu Fabra (UPF), Barcelona, Spain; Department of Psychiatry and Legal Medicine, Universitat Autònoma de Barcelona (UAB), Barcelona, Spain; Psychiatric Department, Hospital de la Santa Creu i Sant Pau, Barcelona, Spain; Sant Pau Mental Health, Institut de Recerca Sant Pau (IR Sant Pau), Barcelona, Spain; Instituto de Investigación Sanitaria del Hospital Universitario de La Princesa, IIS Princesa, Madrid, Spain; Department of Psychiatry, Universidad Autónoma de Madrid, Madrid, Spain; Facultad de Medicina, Universidad Complutense de Madrid (UCM), Madrid, Spain; Instituto de Investigación 12 de Octubre (i+12), Department of Psychiatry, Hospital Universitario 12 de Octubre, Madrid, Spain; Departmento de Medicina y Especialidades Médicas, Universidad de Alcalá, Alcalá de Henares, Madrid, Spain; Instituto Ramón y Cajal de Investigación Sanitaria (IRYCIS), Madrid, Spain; Departmento de Psiquiatría y Salud Mental, Hospital Universitario Infanta Leonor, Madrid, Spain; Departamento de Medicina Clínica, Universidad Miguel Hernández Alicante, Spain; Servicio de Psiquiatría, Hospital Universitario de San Juan Alicante, Spain; Hospital Clínico Universitario de Valencia, Fundación Investigación Hospital Clínico de Valencia, INCLIVA, Valencia, Spain; Department of Medicine, University of Valencia, Valencia, Spain; Department of Medicine, University CEU-UCH, Valencia, Spain; Servicio de Psiquiatría, Hospital Clínico Universitario, Universidad de Salamanca, Instituto de Investigación Biomédica de Salamanca (IBSAL), Salamanca, Spain; Servicio de Psiquiatría, Hospital Universitario Ramón y Cajal, IRYCIS, Madrid, Spain; Parc sanitari Sant Joan de Déu, Institut de Recerca Sant Joan de Déu, Hospitalet de Llobregat, Spain; Servicio de Psiquiatría, Hospital Universitario del Sureste, Madrid, Spain; Department Psychiatry, Hospital Universitario Alava, Vitoria, Spain; Mental Health and Addiction Services, Hospital Universitari de Santa Maria de Lleida, Lleida, Spain; Department of Psychiatry and Psychology, Hospital Clinic of Barcelona, Barcelona, Spain; Clinical Sciences Department, University of Barcelona (UB), Barcelona, Spain; Bellvitge University Hospital – ICS, Psychiatry, Bellvitge Biomedical Research Institute IDIBELL, Neurosciences Group-Psychiatry and Mental Health, L’Hospitalet de Llobregat, Spain; Institute of Health Research of Aragon (IIS Aragón), Miguel Servet University Hospital, Zaragoza, Spain; Research Network on Chronicity, Primary Care and Health Promotion (RICAPPS), Zaragoza, Spain; Psychiatry Department, Faculty of Medicine, University of Zaragoza, Zaragoza, Spain; Centro de Investigación Biomédica en Red (CIBERSAM), Instituto de Salud Carlos III, Madrid, Spain

**Keywords:** Major depressive disorder, treatment resistant depression, selective serotonin reuptake inhibitors, antidepressants, psychotherapy

## Abstract

**Background:**

Selective serotonin reuptake inhibitors (SSRIs) are the first-line treatment for major depressive disorder (MDD), but initial outcomes can be modest.

**Aims:**

To compare SSRI dose optimisation with four alternative second-line strategies in MDD patients unresponsive to an SSRI.

**Method:**

Of 257 participants, 51 were randomised to SSRI dose optimisation (SSRI-Opt), 46 to lithium augmentation (SSRI+Li), 48 to nortriptyline combination (SSRI+NTP), 55 to switch to venlafaxine (VEN) and 57 to problem-solving therapy (SSRI+PST). Primary outcomes were week-6 response/remission rates, assessed by blinded evaluators using the 17-item Hamilton Depression Rating Scale (HDRS-17). Changes in HDRS-17 scores, global improvement and safety outcomes were also explored. EudraCT No. 2007-002130-11.

**Results:**

Alternative second-line strategies led to higher response (28.2% *v.* 14.3%, odds ratio = 2.36 [95% CI 1.0–5.6], *p* = 0.05) and remission (16.9% *v.* 12.2%, odds ratio = 1.46, [95% CI 0.57–3.71], *p* = 0.27) rates, with greater HDRS-17 score reductions (−2.6 [95% CI −4.9 to −0.4], *p* = 0.021]) than SSRI-Opt. Significant/marginally significant effects were only observed in both response rates and HDRS-17 decreases for VEN (odds ratio = 2.53 [95% CI 0.94–6.80], *p* = 0.067; HDRS-17 difference: −2.7 [95% CI −5.5 to 0.0], *p* = 0.054) and for SSRI+PST (odds ratio = 2.46 [95% CI 0.92 to 6.62], *p* = 0.074; HDRS-17 difference: −3.1 [95% CI −5.8 to −0.3], *p* = 0.032). The SSRI+PST group reported the fewest adverse effects, while SSRI+NTP experienced the most (28.1% *v.* 75%; *p* < 0.01), largely mild.

**Conclusions:**

Patients with MDD and insufficient response to SSRIs would benefit from any other second-line strategy aside from dose optimisation. With limited statistical power, switching to venlafaxine and adding psychotherapy yielded the most consistent results in the DEPRE'5 study.

Selective serotonin reuptake inhibitors (SSRIs) are typically the first-line pharmacological options for major depressive disorder (MDD), but their benefits can be initially modest.^1–4^ An early treatment modification seems crucial since the failure or success of a second-attempt treatment is strongly related to long-term outcomes.^5^ Clinical guidelines suggest various strategies after an initial inadequate response to SSRIs, that is, dose optimisation, switching to another antidepressant (preferably to a different pharmacological class), augmentation with a non-antidepressant agent (such as lithium), combination with another antidepressant with a complementary mechanism of action (such as nortriptyline) or adding psychotherapy.^5–7^ Although controversial,^8,9^ SSRI dose optimisation is still one of the preferred initial steps in clinical practice.^10^ Randomised clinical trials have examined the efficacy of many of these strategies with varied results, but virtually none have compared them simultaneously. The most extensive study to investigate the efficacy of different treatment approaches is the STAR*D, which enrolled more than 1200 patients with MDD who eventually failed to respond to the initial SSRI.^11,12^ Patients were then assigned to various switching, combination or augmentation strategies in parallel and sequential steps using an equipoise-stratified randomisation design that considered the patient’s preferences, with no control arm and self-rated measures as primary outcomes. Despite its significant contributions, several methodological aspects have been criticised.^12,13^ In this pragmatic, assessor-blinded, randomised trial (the DEPRE’5 study), we evaluated the most effective strategy for patients with MDD who had an inadequate response to the initial SSRI, a critical clinical point. We compared SSRI dose optimisation (control arm) with SSRIs plus lithium augmentation, SSRIs plus nortriptyline, switching to venlafaxine and SSRIs plus focused problem-solving psychotherapy. We aimed to address two questions: (a) is SSRI dose optimisation a worthless second-line strategy after a failed SSRI? (b) Is any particular second-line strategy more effective than dose optimisation?

## Method

### Trial design

**Fig. 1:**
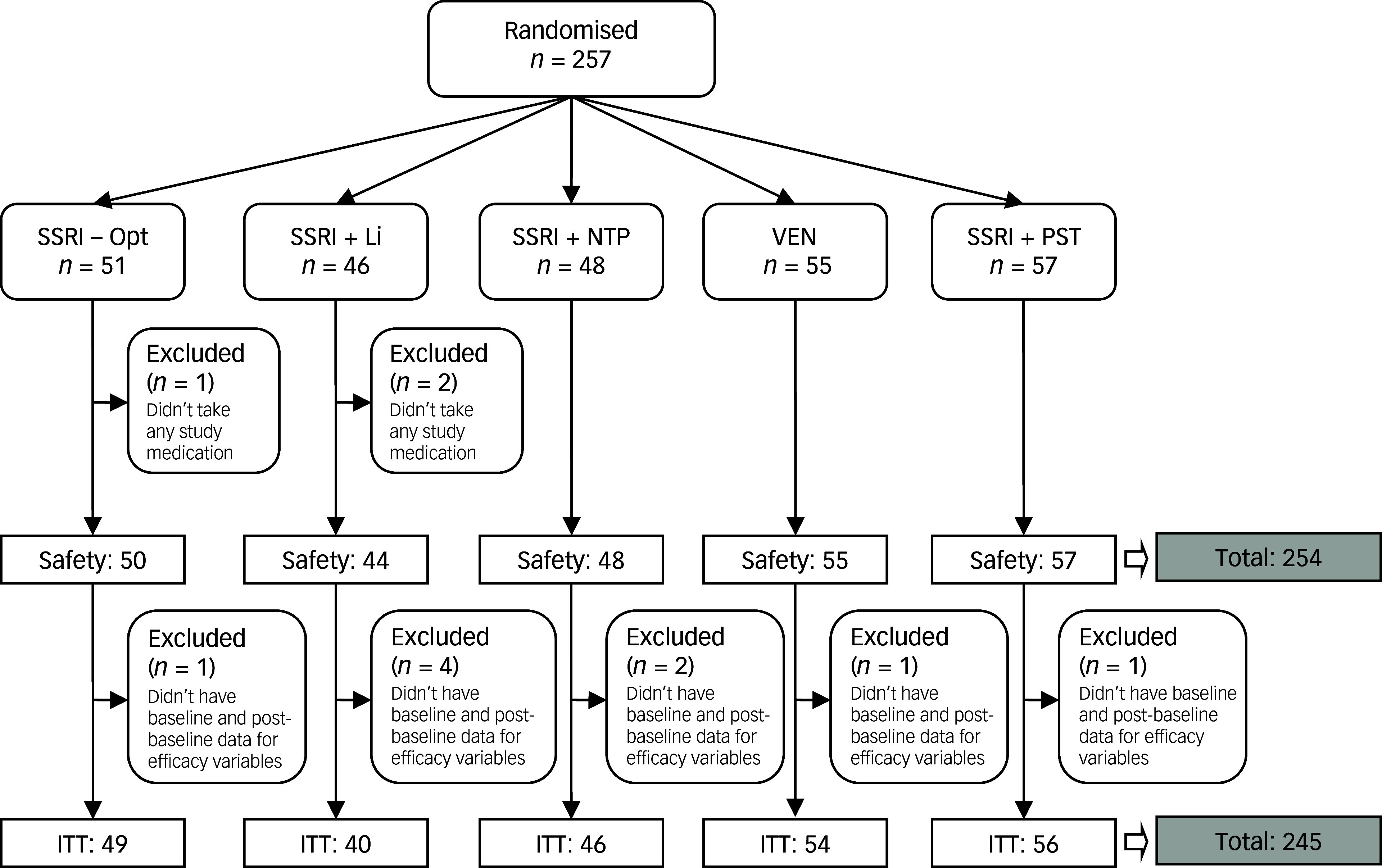
Flow diagram for study participants. SSRI, serotonin reuptake inhibitor; Opt, dose optimisation; Li, lithium; NTP, nortriptyline; PST, problem-solving therapy; VEN, venlafaxine; ITT, intention to treat.

This study adhered to the Consolidated Standards of Reporting Trials (CONSORT; Fig. 1) guidelines.^14^ A five-arm, multicentre, parallel, pragmatic, assessor-blinded, randomised clinical trial for patients with MDD and an insufficient response to SSRIs was designed and conducted at specialised mental health services in ten Spanish hospitals between 2008 and 2012 (the first participant’s first visit was on 16 October 2008).

The authors assert that all procedures contributing to this work comply with the ethical standards of the relevant national and institutional committees on human experimentation and with the Helsinki Declaration of 1975, as revised in 2013. All procedures involving human participants/patients were approved by the Ethical Committee of each site, and written informed consent was obtained before any study procedure (EudraCT registration; 2007-002130-11: https://www.clinicaltrialsregister.eu/ctr-search/trial/2007-002130-11/ES).

### Participants

Participants from both genders, aged between 18 and 75 years and receiving in-patient or out-patient treatment, were eligible providing that they (a) met the criteria for MDD (DSM-IV-TR criteria),^15^ (b) presented an insufficient response to appropriate doses of a SSRI after a minimum of 6 weeks, having a score ≥14 on the 17-item Hamilton Depression Rating Scale (HDRS-17),^16^ (c) were using an accepted contraception method (if women of childbearing potential) and (d) were capable of giving consent.

Exclusion was determined by (a) pregnancy or breastfeeding, (b) HDRS-17 suicide-item score ≥3, (c) severe organic disease, (d) delusions or hallucinations, (e) currently meeting criteria for substance and alcohol misuse or dependence, (f) use of any psychopharmacological agents, other than prescribed benzodiazepines and SSRIs, (g) structured psychotherapeutic treatment and (h) patients with a history of non-response, side-effects or contraindications for any of the study drugs.

### Randomisation and blinding

Patients were randomised to one of five groups: (a) SSRI dose optimisation (SSRI-Opt), (b) lithium augmentation (SSRI + Li), (c) SSRI + nortriptyline (SSRI + NTP), (d) switch to venlafaxine (VEN) or (e) SSRI + problem-solving therapy (SSRI + PST). Randomisation was performed by a contract research organisation (CRO). A single randomisation list was obtained using the SAS Software (v.9.1.3; SAS Institute Inc., Cary, NC, USA). Assessments were at baseline and weeks 1, 2, 4 and 6 post-randomisation. Outcome assessors were blinded to group allocation. The allocation ratio was 1:1:1:1:1. One of 20 lists generated to ensure blinding was selected for the study.

### Treatment arms

*SSRI-Opt:* The SSRI dose was increased up to the maximum dose approved by the European Medicines Agency (EMA): 60 mg/day for fluoxetine, 50 mg/day for paroxetine, 200 mg/day for sertraline, 60 mg/day for citalopram, 20 mg/day for escitalopram and 300 mg/day for fluvoxamine. Higher doses were allowed if deemed necessary and well tolerated.

*SSRI + Li:* Lithium was started at 400 mg daily. Psychiatrists were encouraged to modify the dose (during weeks 1 and 2) to achieve therapeutic levels between 0.5 and 0.8 mmol/l.

*SSRI + NTP:* The starting dose of nortriptyline was 25 mg daily, which increased to 50–75 mg during weeks 1 and 2 and remained stable.

*VEN:* The starting dose of extended-release venlafaxine was 75 mg daily, increasing to 150 mg per day during week 1 and reaching a final maximum dose of 225–300 mg/day. Simultaneously, the SSRI dose was gradually tapered down during week 1.

*SSRI + PST:* This protocol consisted of structured 30-min psychotherapy sessions over 6 weeks. A detailed manual was prepared to guarantee protocol adherence. Two random sessions per therapist were videotaped and supervised.

Flexible dosing was allowed based on clinical judgement to reflect routine clinical practice better. If benzodiazepines were present, their doses remained constant during the study. In those groups where SSRIs were maintained, doses remained constant.

### Outcome and measures

Primary outcomes were response (≥50% decrease from baseline in HDRS-17 score) and remission (HDRS-17 score <8) rates at the end-point (week 6). Secondary outcomes were changes in HDRS-17 scores from baseline and the Clinical Global Impression-Improvement (CGI-I) scale.^17^

Medication adherence was assessed at each visit by pill count for lithium, nortriptyline and venlafaxine and by verbal report for SSRIs. Lithium plasma levels were obtained at weeks two and six and as needed. Safety assessments included vital signs, physical examination, weight and height, blood tests and pregnancy tests at baseline and study end. Adverse events were monitored throughout the study and coded using the Medical Dictionary for Regulatory Activities (MedDRA) system (v.15.1).

### Sample size estimation

Previous studies^3,18^ indicate that approximately 20% of patients who show no response to first-line SSRIs achieve remission rates after a second-line treatment. Based on 80% power to detect 23% of differences between control (SSRI-Opt) and each of the other groups, a bilateral significance level of 0.05 and assuming an attrition rate of 10%, the required sample was 99 participants per group (495 in total). A slower-than-expected recruitment rate in some sites led to the study’s premature termination, not achieving the targeted sample size.

### Statistical analysis

Baseline characteristics were compared using the chi-squared test, Fisher’s exact test or one-way analysis of variance (ANOVA) as needed. We first assessed SSRI dose optimisation against all other second-line strategies. Then, we compared efficacy and safety outcomes between SSRI-Opt and each individual second-line strategy. Fisher’s exact test evaluated primary outcomes, safety and adherence to treatment. Results were expressed as odds ratios and absolute risk differences with 95% confidence intervals. Mixed models for repeated measures (MMRMs) were used for secondary outcomes, using rank transformation for ordinal variables. Subject and error were random effects, and treatment group, visit and treatment-group-by-visit interaction fixed effects, and baseline values were entered as covariates. The within-patient variance–covariance matrix was assumed to be unstructured. Treatment effects were estimated using least square means (LSM) and a 95% confidence interval.

Baseline characteristics and primary outcomes were analysed on the intention-to-treat (ITT) population, which included all randomised subjects with baseline efficacy data and at least one post-baseline efficacy measurement. The safety analysis included all randomised subjects. Missing data for primary variables were treated using the last observation carried forward (LOCF). Basal-observation-carried-forward (BOCF) and available-data-only (ADO) approaches were used for sensitivity analyses.

## Results

### Participants’ baseline characteristics

A total of 257 participants were randomised (245 included in the ITT set and 254 in the safety set): 51 were allocated to SSRI-Opt, 46 to SSRI + Li, 48 to SSRI + NTP, 55 to VEN and 57 to SSRI + PST (Fig. 1). Demographic and clinical baseline characteristics did not differ among groups (Table 1).

**Table 1:** Demographic and baseline clinical features

Feature	Category/unit	Treatment group											
		SSRI-Opt *N* = 49		SSRI + Li *N* = 40		SSRI + NTP *N* = 46		SSRI + PST *N* = 56		VEN *N* = 54		Total *N* = 245	
		Mean	s.d.	Mean	s.d.	Mean	s.d.	Mean	s.d.	Mean	s.d.	Mean	s.d.
Age	Years	49.15	12.43	49.38	11.4	46.96	13.52	48.47	12.4	52.54	11.88	49.37	12.4
		*N*	%	*N*	%	*N*	%	*N*	%	*N*	%	*N*	%
Gender	Male	13	27.1	10	25.6	12	27.3	19	33.9	12	23.1	66	27.6
	Female	35	72.9	29	74.4	32	72.7	37	66.1	40	76.9	173	72.4
Marital status	Single	12	24.5	11	27.5	11	23.9	10	17.9	11	20.4	55	22.4
	Married/cohabitating	26	53.1	23	57.5	22	47.8	33	58.9	27	50.0	131	53.5
	Divorced/separated	7	14.3	5	12.5	8	17.4	9	16.1	11	20.4	40	16.3
	Widowed	4	8.2	1	2.5	5	10.9	4	7.1	5	9.3	19	7.8
Educational level	No formal education	2	4.2	3	7.5	5	11.1	3	5.4	2	3.8	15	6.2
	Primary education	20	41.7	14	35.0	18	40.0	19	33.9	22	41.5	93	38.4
	Secondary education	10	20.8	13	32.5	11	24.4	21	37.5	17	32.1	72	29.8
	University degree	16	33.3	10	25.0	11	24.4	13	23.2	12	22.6	62	25.6
Employment status	Employed or student	15	30.6	11	27.5	13	28.3	17	30.4	21	40.4	77	31.7
	On sick leave	19	38.8	16	40	18	39.1	22	39.3	15	28.8	90	37.4
	Unemployed	9	18.4	8	20	7	15.2	6	10.7	6	11.5	36	40.3
	Retired	6	12.2	5	12.5	8	17.4	11	19.7	10	19.2	40	18.1
		Mean	s.d.	Mean	s.d.	Mean	s.d.	Mean	s.d.	Mean	s.d.	Mean	s.d.
Number of previous episodes		2	1.35	2.08	2.36	1.7	1.11	2.07	1.88	1.94	1.28	1.96	1.62
		*N*	%	*N*	%	*N*	%	*N*	%	*N*	%	*N*	%
Recurrent depression		28	57.1	24	60.0	27	58.7	27	48.2	30	55.6	136	55.5
Duration of current episode													
2 weeks to 1 month		1	2.0	0	0.0	0	0.0	0	0.0	1	1.9	2	0.8
1–6 months		18	36.7	16	40.0	23	50.0	20	35.7	18	34.0	95	38.9
7–12 months		19	38.8	9	22.5	15	32.6	19	33.9	13	24.5	75	30.7
1–2 years		10	20.4	9	22.5	4	8.7	10	17.9	13	24.5	46	18.9
More than 2 years		1	2.0	6	15.0	4	8.7	7	12.5	8	15.1	26	10.7
Psychiatric disorder in first-degree family member		24	50.0	19	47.5	23	51.1	30	53.6	22	40.7	118	48.6
Ever attempted suicide		4	8.2	5	12.8	1	2.2	9	16.4	4	7.7	23	9.5
Other past psychiatric disorders		6	12.5	5	12.8	8	17.4	12	21.8	7	13.5	38	15.8
Treatment strategies in previous depressive episodes													
Augmentation therapy		1	2.0	0	0.0	0	0.0	2	3.6	0	0.0	3	1.2
Combination therapy		2	4.1	1	2.5	3	6.5	3	5.4	1	1.9	10	4.1
Psychotherapy		10	20.4	4	10.0	6	13.0	6	10.7	5	9.3	3	12.7
Electroconvulsive therapy		0	0	0	0.0	0	0.0	1	1.8	0	0.0	1	0.4

SSRI, serotonin reuptake inhibitor; Opt, dose optimisation; Li, lithium; NTP, nortriptyline; PST, problem-solving therapy; VEN, venlafaxine.

Missing data in certain features may change the percentage calculation denominator, affecting comparisons with the total count.

Before randomisation, 30% were on escitalopram, 20.8% on fluoxetine, 22% on paroxetine, 20% on citalopram, 6.8% on sertraline and 0.4% on fluvoxamine. Mean daily doses of SSRIs (as fluoxetine equivalents) increased from 33.2 to 55 mg for the SSRI-Opt group and remained constant for the other groups: 31.7mg for SSRI + Li, 34.6 mg for SSRI + NTP and 30.7 mg for SSRI + PST (see Supplementary Tables S1 and S2). At the study’s end, mean daily doses were 493.8 mg lithium (mean plasma level 0.54 mM), 51.3 mg nortriptyline and 151.4 mg VEN (75.9% received at least 150 mg, 22.2% received higher doses).

Medication adherence differed significantly among groups (*p* < 0.001): participants in the SSRI + Li group had the lowest adherence (38.6%) compared to the others (98.2% in SSRI + PST, 96.4% in VEN, 72% in SSRI-Opt and 68.8% in SSRI + NTP). Note that any deviation from the prescribed regimen in at least two visits was considered low adherence. Participants attended an average of 7.1 PST sessions (s.d. = 1.9), and 89.5% completed the intervention. Thirty-three patients (13.5% of the ITT sample) withdrew without significant group differences. The main reasons included lack of efficacy (SSRI, SSRI + PST), adverse events (SSRI + Li, SSRI + NTP) and patient choice (VEN).

### SSRI dose optimisation versus second-line strategies

**Fig. 2:**
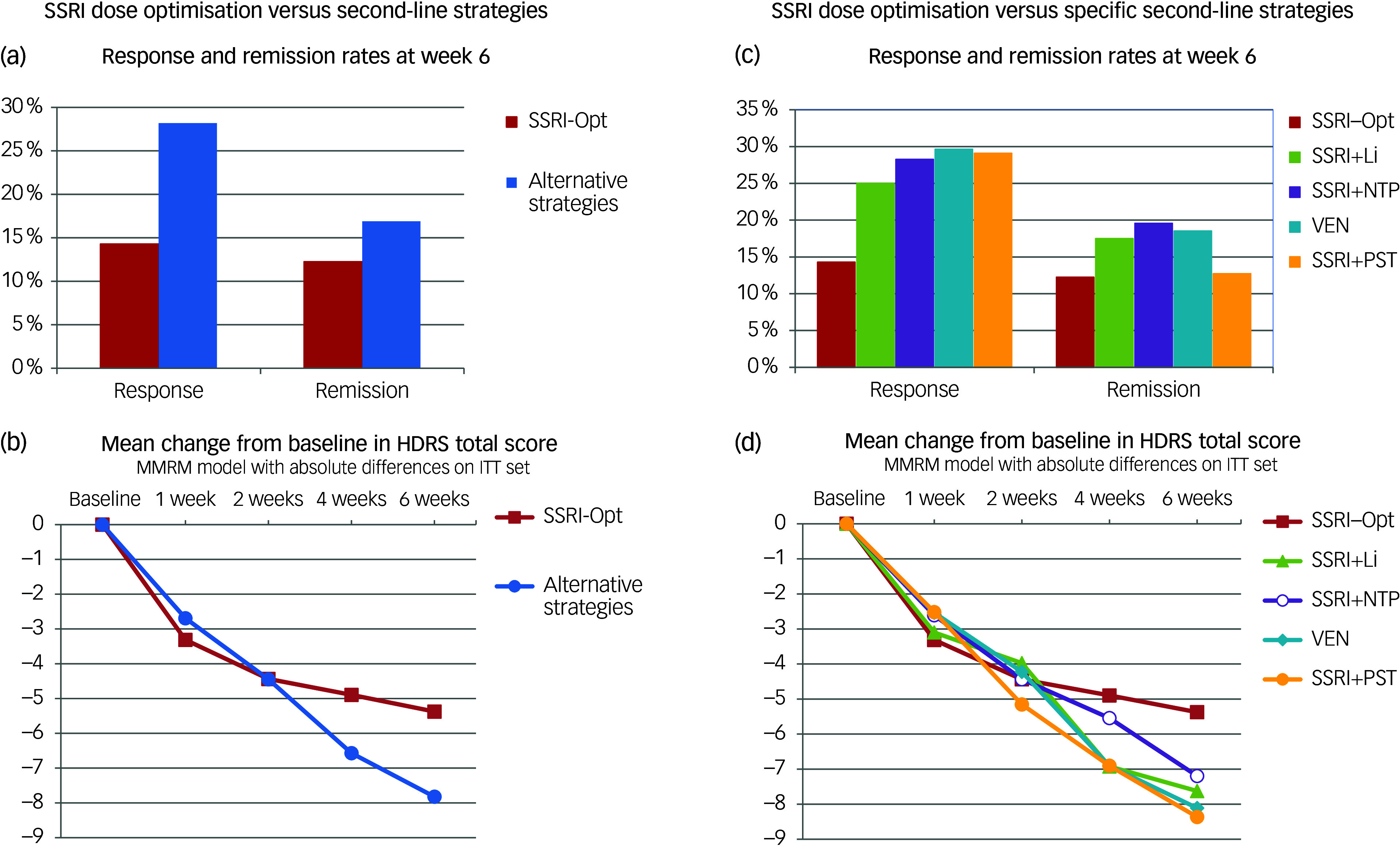
Response/remission rates at week 6 and mean Hamilton Depression Rating Scale (HDRS) score change from baseline. SSRI, serotonin reuptake inhibitor; Opt, dose optimisation; Li, lithium; NTP, nortriptyline; PST, problem-solving therapy; VEN, venlafaxine; ITT, intention to treat; MMRM, mixed model for repeated measures.

Patients receiving second-line treatments (SSRI + Li, SSRI + NTP, VEN or SSRI + PST) had higher response rates (28.2%) than those on SSRI-Opt (14.3%, odds ratio = 2.36, 95% CI = 1.0–5.6; *p* = 0.05; Fig. 2(a)). Dose optimisation significantly reduced the likelihood of response by 13.9% (95% CI = 12.6, 25.6; *p* = 0.0193). The alternative strategies had a higher remission rate (16.9%) compared to the SSRI-Opt group (12.2%), but the difference was not significant (odds ratio = 1.46, 95% CI = 0.57–3.71; *p* = 0.27).

MMRM analysis revealed a treatment-by-assessment point effect (*p* = 0.023), showing a significant difference in the mean HDRS-17 change from baseline between patients who received an alternative second-line strategy and those on SSRI dose optimisation alone (HDRS-17 reduction difference = −2.6, 95% CI = −4.9 to −0.4; *p* = 0.021; Fig. 2(b)). Alternative second-line strategies were associated with more significant improvements in CGI-I scores: 49.2% showed ‘much improvement’ compared to 30.6% in the SSRI group (odds ratio = 2.20, 95% CI = 1.12, 4.29; *p* = 0.02).

### SSRI dose optimisation versus specific second-line strategies

There were no significant differences in response and remission rates when comparing the SSRI group with any other second-line therapeutic strategy (Fig. 2(c)). The likelihood of response was twice for each of the four second-line strategies compared to SSRI alone. However, differences only reach a marginally significant effect for the VEN and SSRI + PST groups: SSRI + Li versus SSRI, odds ratio = 2.00 (95% CI = 0.68, 5.85; *p* = 0.206); SSRI + NTP versus SSRI, odds ratio = 2.36 (95% CI = 0.85, 6.59; *p* = 0.100); VEN versus SSRI, odds ratio = 2.53 (95% CI = 0.94, 6.80; *p* = 0.067); SSRI + PST versus SSRI, odds ratio = 2.46 (95% CI = 0.92, 6.62; *p* = 0.074; Fig. 2(d)).

A significant reduction in total HDRS-17 scores was found for the SSRI + PST group compared to the SSRI-Opt group (−3.1, 95% CI = −5.8, −0.3; *p* = 0.032). Marginally significant reductions were also observed for the VEN (−2.7, 95% CI = −5.5, 0.0; *p* = 0.054) and SSRI + Li groups (−2.6, 95% CI = −5.6, 0.4, *p* = 0.086). Sensitivity analyses using MMRMs with the BOCF and ADO approaches revealed significant differences favouring SSRI + PST (*p* = 0.028 and *p* = 0.032, respectively), VEN (*p* = 0.033 and *p* = 0.016, respectively) and SSRI + Li (*p* = 0.066 and *p* = 0.027, respectively).

The number of patients achieving ‘much improvement’ or better rating on the CGI-I scale at the study end was significantly higher in the SSRI + NTP group (51.1%; odds ratio = 2.37, 95% CI = 1.02, 5.51; *p* = 0.04) and in the VEN group (50.9%; odds ratio = 2.35, 95% CI = 1.05, 5.30; *p* = 0.04) compared to the SSRI-Opt group (30.6%). Participants receiving SSRI + PST (47.3%; odds ratio = 2.03, 95% CI = 0.91, 4.55; *p* = 0.08) and SSRI + Li (47.5%; odds ratio = 2.05, 95% CI = 0.86, 4.89; *p* = 0.10) showed a more significant improvement on the CGI-I scale, although not statistically significant.

### Safety outcomes

**Fig. 3:**
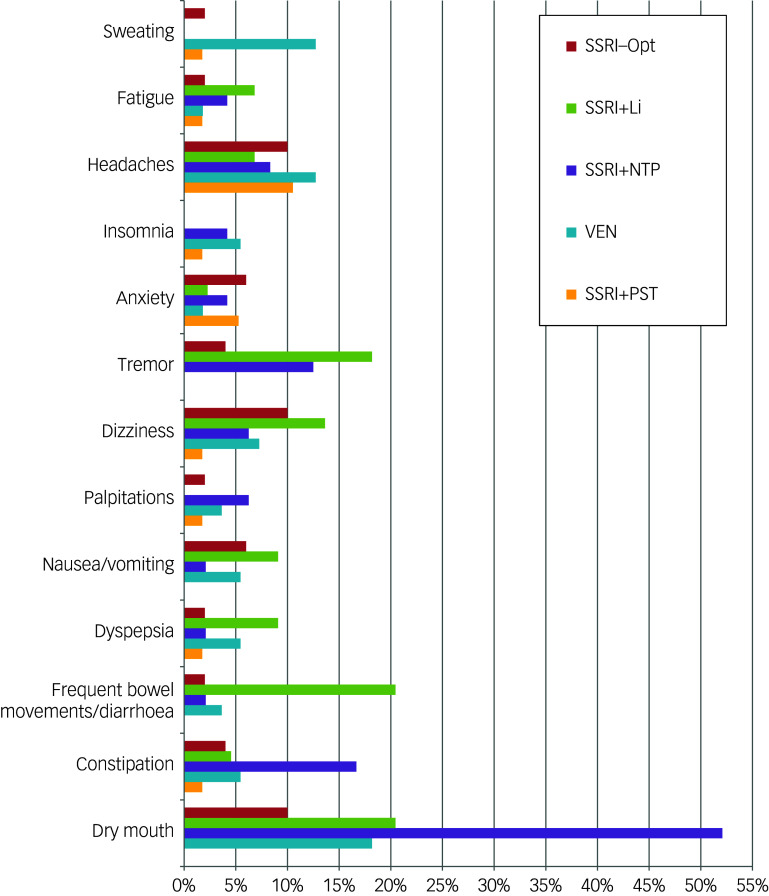
Incidence of adverse effects occurring in more than 5% of patients in each treatment group. SSRI, serotonin reuptake inhibitor; Opt, dose optimisation; Li, lithium; NTP, nortriptyline; PST, problem-solving therapy; VEN, venlafaxine.

The SSRI + PST group reported the fewest treatment-emergent adverse events, while the SSRI + NTP group experienced the most (28.1% *v.* 75%, respectively; *p* < 0.01). Largely, adverse events were mild (81.2%) or moderate (17.8%). Compared to others, dry mouth was more frequent in the SSRI + NTP group (*p* < 0.01), and bowel movements and diarrhoea were more frequent in the SSRI + Li group (*p* ≤ 0.01). Compared to the SSRI-Opt group, the SSRI + NTP group presented more constipation (*p* = 0.05), and the SSRI + Li group was associated with more tremors and gastrointestinal complaints (*p* = 0.04). Sweating was associated with venlafaxine use (*p* = 0.06). One participant in the SSRI + PST group presented a low-lethality suicide attempt (Fig. 3).

## Discussion

The DEPRE’5 study, a pragmatic multicentre five-arm randomised controlled trial, assessed the efficacy of different treatment strategies for patients with MDD after an unsuccessful SSRI treatment. SSRI dose optimisation resulted in the lowest response rates, suggesting other alternatives should be considered. No significant differences in primary outcomes were found when comparing each strategy individually with the SSRI optimisation arm. However, the lower-than-planned recruitment rate could explain the lack of greater benefits of some of the explored treatments. Switching to venlafaxine, combining SSRIs with psychotherapy and, to a lesser extent, lithium augmentation offered better outcomes in terms of response rate, change in depressive symptoms and/or global improvement compared to dose optimisation. All conditions were safe, but patients receiving psychotherapy experienced fewer adverse events.

Although antidepressant dose optimisation is debated,^8,9^ real-world prescription patterns show that it is still a common approach for managing depressive episodes.^10^ Surprisingly, SSRI monotherapy remains popular even after a second treatment failure, with long delays before exploring alternative strategies such as augmentation or combination.^19,20^ While higher SSRI doses may boost efficacy,^8^ we found that using a second-line approach – either expanding the mechanism of action or adding psychotherapeutic interventions – doubled the likelihood of response in the case of inadequate SSRI response.

Switching to venlafaxine showed potential as one of the more beneficial second-line strategies in our sample. While somewhat controversial, previous literature supports the increased effectiveness of dual-acting antidepressants in patients with severe depression or inadequate SSRI response.^21,22^ In this trial, most patients on venlafaxine received at least 150 mg/day, a dose modulating both monoaminergic pathways without compromising tolerability.^9^

Lithium augmentation showed a trend towards greater symptom reduction at the end-point. However, the small sample size may have limited the ability to demonstrate more robust benefits. Although mean plasma levels were within the desired range, the SSRI + Li group showed lower adherence, which may have also affected the outcomes. Lithium users experienced more tremors and gastrointestinal discomfort but with no differences in drop-out rates. Meta-analyses support the efficacy of lithium augmentation in cases of inadequate antidepressant response, even when combined with SSRIs.^23,24^ Preliminary analyses suggest it may be more cost-effective than other augmentation strategies, such as atypical antipsychotics, with similar acceptability and tolerability.^25^ The low usage rate may be because of clinicians’ inexperience, the need to monitor lithium levels or concerns about its narrow safety range. Despite less promotion and research, lithium remains a long-standing, consistent strategy in MDD. Further research should clarify whether lithium augmentation’s benefits are specific to certain groups of patients.^23,26^

Our results support the utility of PST, a structured and goal-oriented cognitive–behavioural intervention, chosen for its effectiveness, applicability and limited duration, comparable to the other treatment arms.^27^ Beyond the STAR*D study,^28^ limited research has compared the effectiveness of combining psychotherapy with antidepressants in patients with previous treatment failures. Initial evidence suggests that combined treatment may be more effective than single treatments.^7,29^ Although SSRIs relieve some symptoms faster than psychotherapy, the latter, when combined with antidepressants, can be more effective in achieving behavioural activation and positive experiences, crucial aspects for a full recovery. Psychotherapy, alone or combined, is often better accepted by patients with mild to moderate depression. This matches the strong adherence seen in the SSRI + PST group and the lower adverse event rate.

The low remission rates are consistent, albeit slightly lower than those observed in comparable pragmatic studies.^30,31^ In contrast to the STAR*D study, which relied on an equipoise-stratified randomisation scheme, our participants were randomised using a classic randomisation approach regardless of the patient and clinician preferences, and raters were blinded. These factors influence expectations, treatment adherence and perceived efficacy.^32^ In clinical practice, choosing a particular second-line strategy relies on the degree of the previous antidepressant response (i.e. optimisation, augmentation or combination in the case of a partial response versus switching in the case of null response) or the type of predominant symptoms.^33–35^ Unfortunately, we could not assess differences between groups based on the previous dose or the degree of response to the initial SSRI. The presence of specific stressful life events or maladaptive coping strategies could make psychotherapy the most appropriate option. Further research should identify clinical and neurobiological features that could moderate treatment responses to these alternatives, leading to more effective therapeutic decisions.

The duration of the depressive episode is one of the factors most clearly associated with the risk of persistence and treatment failure.^36^ In our study, almost 30% of the participants had an episode lasting over a year, over half had experienced previous episodes, 48% had at least one first-degree relative with a psychiatric history and nearly 10% had a lifetime history of suicidal behaviour. These factors could have influenced our patients’ likelihood of remission within 6 weeks.^36^

Judging by the symptom progression over the follow-up period, some participants may have achieved remission with the assigned treatment beyond that end-point. The VAST-D study, which tested the efficacy of different strategies in a sample of US veterans with MDD unresponsive to a first-line antidepressant,^30^ showed even lower remission rates at week 6 compared to our study, with improvements in the subsequent weeks. Eight to 12 weeks may be necessary to achieve the maximum symptomatic reduction with a new line of antidepressant treatment, especially after initial treatment failure.^33^ In any case, our results support that the failure of one treatment reduces the odds of remission^3^ and underscores the need to use other strategies than dose optimisation.

Several limitations have to be acknowledged. First, the low recruitment rate resulted in a lower-than-expected sample size and compromised our statistical power. Recruitment was challenging because participating sites often encountered participants who had been treated with multiple therapeutic lines at the initial assessment. Site was not included as a covariate, although the centres’ homogeneity, standardised protocols and centralised randomisation likely mitigated site-related variability. In addition, the study’s lack of a double-blind design and the use of the LOCF approach for imputing missing data may have influenced our findings. Since depressive symptoms tend to improve over time, a more extended follow-up period would have been needed. The degree of prior SSRI response, as well as clinical and patient’s expectations are rarely assessed in similar trials but may influence real-world treatment choices and outcomes. For some, optimising the dose of an ineffective antidepressant can be discouraging; for others, concerns about trying a new therapeutic option may make adjusting the dose of a known antidepressant preferable. Baseline doses of SSRIs were mostly appropriate but not maximal, supporting flexible adjustments in the SSRI-Opt group based on prior response, tolerability and perceived efficacy. The neutral way in which participants were informed about the common use and potential benefits of each arm, along with randomised treatment allocation and blinded assessment, may have helped mitigate these effects. Finally, other treatment alternatives (i.e. atypical antipsychotic augmentation, SSRI with α2-antagonist combination or other psychotherapeutic approaches) were not explored.^24,37^

In conclusion, patients with MDD who do not respond to SSRIs may benefit from exploring alternative second-line strategies beyond simply increasing doses. With limited statistical power, switching to venlafaxine and adding psychotherapy yielded more consistent results compared to dose optimisation.

## Supporting information

Pérez et al. supplementary material Pérez et al. supplementary material

## Data Availability

The data supporting this study’s findings are available from the corresponding author, M.E., upon reasonable request.

## Supplementary material

The supplementary material can be found at https://doi.org/10.1192/bjp.2025.13
