# Supplementary material for: The DEPRE’5 study: pragmatic, multicentre, five-arm, parallel-group randomised controlled trial with blinded assessment to compare treatment strategies in major depression after a failed selective serotonin reuptake inhibitor treatment
Source: Br J Psychiatry. 2025 Jun 18;227(5):748–55. doi: 10.1192/bjp.2025.13 (PMC12550662; doi:10.1192/bjp.2025.13)
Supplement: Pérez et al. supplementary material [file S0007125025000133sup001.docx]

Supplementary material

Contents:

**Supplementary Table S1**

**Supplementary Table S2**

**Supplementary references**

Table S1

*Type of SSRI and mean daily dose of this SSRI at baseline, by group*

| **Type of SSRI** | **Treatment group** | | | | | | | | | |
| --- | --- | --- | --- | --- | --- | --- | --- | --- | --- | --- |
|  | **SSRI-Opt**  **N=51** | | **SSRI + Li**  **N=46** | | **SSRI + NTP**  **N=48** | | **VEN**  **N=55** | | **SSRI + PST**  **N=57** | |
|  | **N** | **Mean daily dose**  ± **SD (mg/d)** | **N** | **Mean daily dose**  ± **SD (mg/d)** | **N** | **Mean daily dose**  ± **SD (mg/d)** | **N** | **Mean daily dose**  ± **SD (mg/d)** | **N** | **Mean daily dose**  ± **SD (mg/d)** |
| **Citalopram** | 11 | 28.13 ± 11.32 | 8 | 23.75 ± 5.18 | 6 | 25.83 ± 10.21 | 8 | 25.00 ± 10.69 | 17 | 27.35 ± 11.47 |
| **Escitalopram** | 15 | 19.29 ± 8.96 | 16 | 18.44 ± 9.61 | 21 | 18.57 ± 11.74 | 12 | 15.00 ± 4.26 | 11 | 14.55 ± 4.72 |
| **Fluoxetine** | 11 | 24.55 ± 8.20 | 13 | 26.15 ± 12.61 | 7 | 22.86 ± 12.54 | 9 | 23.33 ± 7.07 | 12 | 29.17 ± 14.43 |
| **Fluvoxamine** | 0 | NA | 0 | NA | 1 | 250.00 ± NA | 0 | NA | 0 | NA |
| **Paroxetine** | 10 | 24.00 ± 6.99 | 8 | 25.00 ± 9.26 | 10 | 25.00 ± 8.50 | 17 | 25.88 ± 7.12 | 10 | 26.00 ± 9.66 |
| **Sertraline** | 2 | 150.00 ± 70.71 | 1 | 100.00 ± NA | 2 | 100.00 ± 0.00 | 6 | 116.67 ± 51.64 | 6 | 100.00 ± 54.77 |
| **TOTAL**  **(Fluoxetine equivalents) ^†^** | 49 | 33.20 ± 13.33 | 46 | 31,77 ± 13.79 | 47 | 34,61 ± 16.96 | 52 | 31,00 ± 10.21 | 56 | 30,72 ± 13.05 |
| Missings **^‡^** | 2 |  | 0 |  | 1 |  | 3 |  | 1 |  |

*Note.* SSRI = serotonin reuptake inhibitor. Opt = Dose Optimization. Li = Lithium. NTP = nortriptyline. VEN = Venlafaxine. PST = Problem-Solving Therapy. Mg = miligrams. SD = Standard deviation. NA = Not applicable.

**^†^** Dose conversion algorithms based on Hayasaka Y, Purgato M, Magni LR. Dose equivalents of antidepressants: evidence-based recommendations from randomized controlled trials. J Affect Disord. 2015;180:179–184 and Furukawa TA, Cipriani A, Cowen PJ, Leucht S, Egger M, Salanti G. Optimal dose of selective serotonin reuptake inhibitors, venlafaxine, and mirtazapine in major depression: a systematic review and dose-response meta-analysis. Lancet Psychiatry. 2019;6:601-609.

**^‡^** These participants belong to the group excluded from the intention-to-treat sample

Table S2

Type of SSRI and mean daily dose at baseline and at the end of the study in SSRI-Opt group

| **Type of SSRI** | **SSRI-Opt group**  **N=51** | | | |
| --- | --- | --- | --- | --- |
|  | **N** | **BASELINE** | **FINAL VISIT** | **Percentage change** |
|  |  | **Mean daily dose**  ± **SD (mg/d)** | **Mean daily dose**  ± **SD (mg/d)** |  |
| **Citalopram** | 11 | 28.13 ± 11.32 | 43.50 ± 14.92 | 54.64% |
| **Escitalopram** | 15 | 19.29 ± 8.96 | 31.67 ± 12.20 | 64.18% |
| **Fluoxetine** | 11 | 24.55 ± 8.20 | 47.27 ± 10.09 | 92.54% |
| **Fluvoxamine** | 0 | NA | NA | NA |
| **Paroxetine** | 10 | 24.00 ± 6.99 | 39.00 ± 7.38 | 62.5% |
| **Sertraline** | 2 | 150.00 ± 70.71 | 225.00 ± 35.36 | 50% |
| **TOTAL**  **(Fluoxetine equivalents)^†^** | 49 | 33.20 ± 13.33 | 55.02 ± 16.27 | 65.72% |
| Missings **^‡^** | 2 |  |  |  |

*Note.* SSRI = serotonin reuptake inhibitor. Opt = Dose Optimization. Mg = miligrams. SD = Standard deviation. NA = Not applicable.

**^†^** Dose conversion algorithms based on Hayasaka Y, Purgato M, Magni LR. Dose equivalents of antidepressants: evidence-based recommendations from randomized controlled trials. J Affect Disord. 2015;180:179–184 and Furukawa TA, Cipriani A, Cowen PJ, Leucht S, Egger M, Salanti G. Optimal dose of selective serotonin reuptake inhibitors, venlafaxine, and mirtazapine in major depression: a systematic review and dose-response meta-analysis. Lancet Psychiatry. 2019;6:601-609.

**^‡^** These participants belong to the group excluded from the intention-to-treat sample

Supplementary references

1. Hayasaka Y, Purgato M, Magni LR. Dose equivalents of antidepressants: evidence-based recommendations from randomized controlled trials. J Affect Disord. 2015;180:179–184
2. Furukawa TA, Cipriani A, Cowen PJ, Leucht S, Egger M, Salanti G. Optimal dose of selective serotonin reuptake inhibitors, venlafaxine, and mirtazapine in major depression: a systematic review and dose-response meta-analysis. Lancet Psychiatry. 2019;6:601-609.
